# Osteoprotegerin as a biomarker of geriatric frailty syndrome

**DOI:** 10.18632/aging.102083

**Published:** 2019-07-17

**Authors:** Alessia Valentini, Maria Assunta Cianfarani, Umberto Tarantino, Nicola Di Daniele, Aldo Bertoli

**Affiliations:** 1 Department of Systems Medicine, University of Rome “Tor Vergata”, 00133 Rome, Italy; 2 Department of Orthopaedics and Traumatology, University of Rome “Tor Vergata”, 00133 Rome, Italy

**Keywords:** frailty, aging, geriatric assessment, biomarkers, OPG

## Abstract

The lack of a univocal definition of frailty, a condition frequently found in the elderly population which is correlated with an increased risk of mortality, has prompted the search for clinical and laboratory parameters associated with this condition. Whereas OPG is a protein involved in different pathophysiological conditions including bone, vascular, immune and tumor disease and studies found a positive linear correlation between OPG and age we hypothesized that it may represent a frailty marker in the elderly.

We conducted an observational study of 172 elderly subjects, with and without hip fracture, including a multidimensional geriatric evaluation and a laboratory evaluation, aimed to evaluate the association between OPG and frailty.

Frailty Score was associated with FT3 and osteoprotegerin (OPG), regardless of fracture event. Excluding subjects with hip fracture, in whom the acute event had a direct effect on bone production of OPG, the Frailty Score showed a linear correlation with circulating levels of osteoprotegerin.

In the elderly, an increase in osteoprotegerin levels may reflect a progressive accumulation of organ damage leading to the development of frailty. The correlation between OPG and Frailty Score found in our study points to its potential use as a biomarker for geriatric frailty syndrome.

## Introduction

The 20th century was characterized worldwide by important demographic changes [1] that contributed to the progressive ageing of the population, especially in industrialized countries [2].

Ageing, a physiological process that involves all organs and apparatuses of an organism, is accompanied by progressive functional alterations that cause a gradual reduction, and eventually loss of, the maintenance capacity of homeostasis, and is associated with the development of frailty [3,4]. Frailty is defined as a biological syndrome characterized by a reduction in the functional reserves of an organism and in its resistance to stressful events [5], derives from a progressive failure of different physiological systems and leads to an increase in vulnerability to stressful events [6] and a reduction in survival [7].

Frailty has been identified as a distinct biological syndrome [6] the development of which contribute to various conditions, such as chronic inflammation and changes in the immune and endocrine systems [5,8,9], and is associated with an increased risk of death [6,10].

To date, there is no universally accepted definition of frailty [11]. This accounts for the difficulties encountered by clinicians and researchers in identifying a reference tool that allows a simple, precise and easily reproducible evaluation of frailty. Among the different tools proposed over the years to identify frailty [12,13], the phenotypic model introduced by Fried et al. [6] is the most widely used [14], although various algorithms based on laboratory tests have also been proposed [15].

According to the phenotypic model of Fried et al., a subject is identified as frail when she/he has at least three of the following characteristics: 1) weight reduction greater than 4.5 kg or greater than 5% of body weight with respect to the usual weight of the subject in a year; 2) muscle weakness, characterized by a reduction in muscle strength of 20% or more compared to the baseline minimum reference value, standardized by sex and body mass index; 3) asthenia and easy fatigue, referred by the subject as a feeling of exhaustion; 4) motor sluggishness, defined by a reduction in walking speed, stratified by sex and height (the subject takes longer than the maximum time established to travel 4.572 m at the walking speed usually attributed to her/him or is unable to walk); 5) reduction in the level of physical activity and weekly energy expenditure, which is less than 383 Kcal / week for men and below 270 Kcal / week for women [6].

In our study, we chose to evaluate frailty using SHARE-FI [16], based on the criteria proposed by LP Fried [6]. This allowed us not only to identify the frailty phenotype but also to quantify the degree of frailty of an individual [16]. Studies seeking to identify biomarkers as possible indicators of frailty have shown that some of these are associated with an increased risk of mortality [17–19].

Osteoprotegerin (OPG) was originally identified as an inhibitor of the Receptor Activator of Nuclear factor Kappa B Ligand (RANKL) in bone tissue [20], but subsequent studies proved that OPG was also a marker of vascular damage, and its plasma concentrations have been correlated, in different populations, with an increased risk of both all-cause mortality and cardiovascular mortality [21–23]. Moreover, OPG is a protein involved in different physiopathologic conditions including bone, vascular, immune and tumor disease [24] and several studies found a positive linear correlation between OPG and age [25–27].

However, no study to date has investigated the relationship between OPG and frailty, a condition that, as already underscored, is able to significantly compromise the maintenance capacity of homeostasis of an organism, with a consequent increase in the risk of death [4,6].

The aim of this study was to evaluate osteoprotegerin as a possible biochemical marker associated with frailty in the elderly.

## RESULTS

The average age of the study population was 79.56 ± 7.20 years and was higher in frail subjects compared to pre-frail and non-frail subjects (ANOVA p<0.0001), as shown in Table 1. However, it did not differ between patients with fracture and patients without fracture. The evaluation of frailty through SHARE-FI required the assessment of muscle grip strength, measured using handgrip dynamometry. As this was not possible to obtain for 12 of the subjects, the data on frailty were only available for 160 subjects (Table 1).

**Table 1:** Characteristics of the study population and laboratory parameters.

	**Frail**	**Pre-Frail**	**Non-Frail**	**p**
Age (years)*	82.00±7.47	78.09±6.11	76.56±6.44	**-**
**Males**	11	9	27	**-**
**Females**	58	34	21	**-**
**Frailty Score**	3.95±0.90	1.38±0.59	0.038±0.62	< 0.0001
**Frailty Score (Females)**	3.87±0.89	1.23±0.52	-0.21±0.43	< 0.0001
**Frailty Score (Males)**	4.39±0.87	1.98±0.43	0.23±0.68	< 0.0001
**BMI (Kg/m^2^)**	26.40±5.58	27.30±5.60	25.53±4.18	NS
**WBC (10^3^/μl)**	8.63±2.85	8.09±2.91	8.23±3.26	NS
**Hemoglobin (g/dl)**	11.64±1.77	12.71±1.65	13.22±1.58	< 0.0001
**Creatinin (mg/dl)**	1.06±1.07	0.90±0.49	1.11±0.75	NS
**Albumin (g/dl)**	2.88±0.64	3.23±0.82	3.43±0.84	< 0.0005
**TSH (μUI/ml)**	2.18±1.61	1.98±1.92	2.03±1.89	NS
**FT4 (ng/dl)**	1.18±0.20	1.21±0.24	1.22±0.18	NS
**FT3 (pg/ml)**	2.22±0.65	2.63±0.63	2.86±0.62	< 0.0001
**OPG (pmol/l)**	9.31±4.00	7.65±3.23	7.09±3.01	< 0.005
**IL-6 (pg/ml)**	102.23±76.67	80.70±95.51	58.43±88.07	< 0.05
**TNF-α (pg/ml)**	12.26±10.53	10.02±6.13	8.48±3.78	< 0.05
**hs-CRP (mg/dl)**	7.97±5.73	5.62±6.06	4.44±5.24	< 0.01
**Cortisol (μg/dl)**	20.03±7.62	16.73±3.98	16.31±5.74	< 0.01
**DHEAS (μg/dl)**	45.56±30.02	49.82±37.39	51.48±36.14	NS
**IGF-1 (ng/ml)**	71.00±40.34	79.27±34.48	95.55±49.63	< 0.05
**IGFBP-3 (μg/ml)**	1.77±0.72	2.05±0.88	2.52±1.01	< 0.005

Data are presented as mean ± standard deviation

As shown in Table 1, frail subjects had lower mean values of hemoglobin, albumin, FT3, IGF-1 and IGFBP-3 and higher mean values of IL-6, cortisol, hs-CRP, TNF-α and OPG, compared to pre-frail and non-frail subjects.

**Figure 1:**
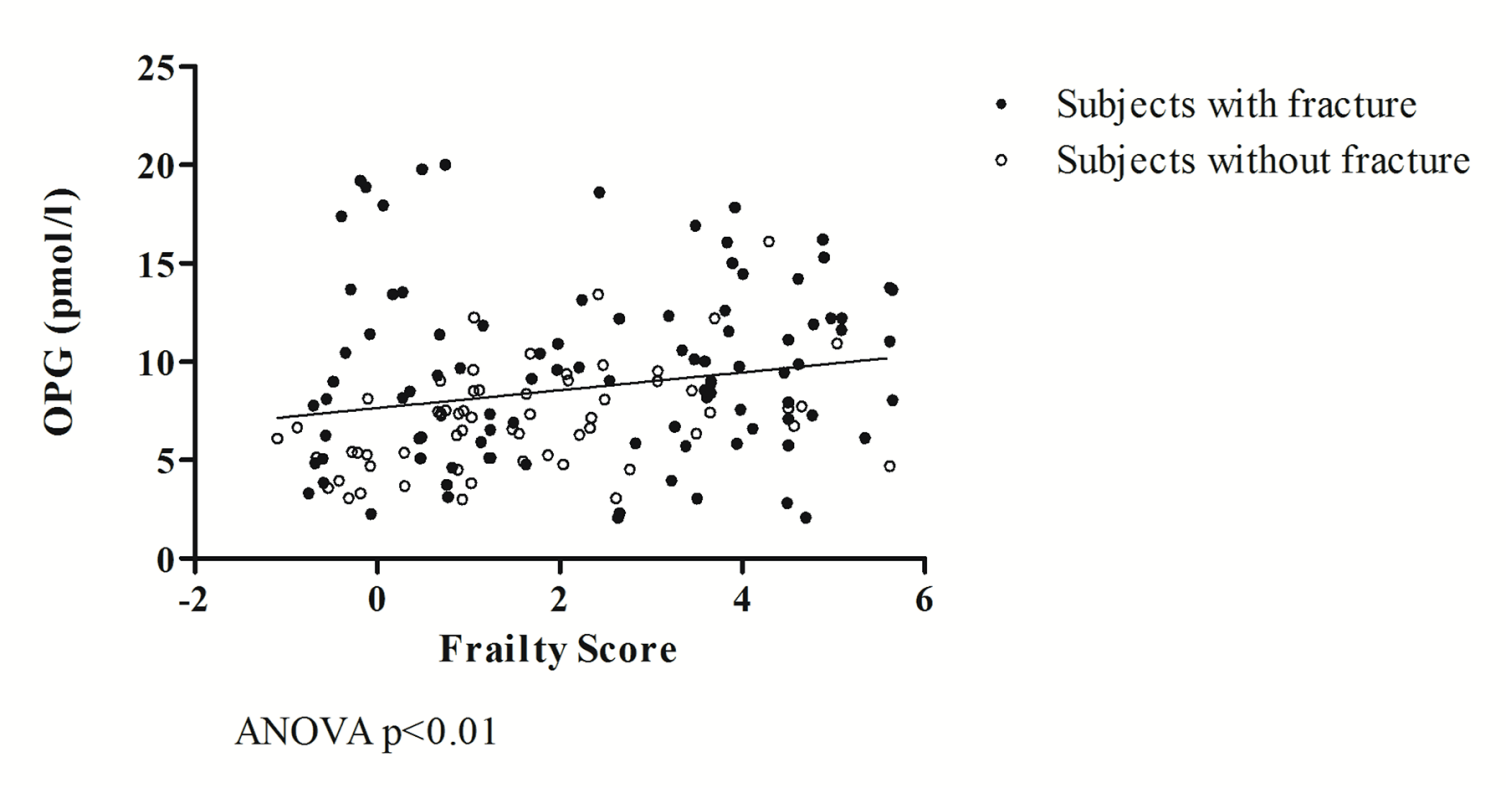
**The relationship between OPG and frailty.**

In a linear regression model (Figure 1) of the entire study population, OPG was directly and significantly correlated with the values of the Frailty Score (ANOVA r=0.208; p<0.01). OPG mean values were 9.59 ± 4.60 in subjects with hip fracture and 7.03 ± 2.58 in subjects without fractures (p<0.001).

A linear correlation was also found between OPG and osteoporosis, evaluated by neck femoral T-score (p<0.001), but not with lumbar vertebral T-score or femoral and vertebral BMD. Furthermore, a correlation exists between OPG and renal function evaluated by means of estimated GFR (p<0.01), but not by plasma creatinine. OPG was also positively correlated with age (p<0.05).

A covariance analysis (ANCOVA) shows how a fracture event influences, in a statistically significant manner, the regression between OPG and Frailty Score (F=14.40; p<0.001), which are closely associated with one another (F =4.32; p<0.05).

**Figure 2:**
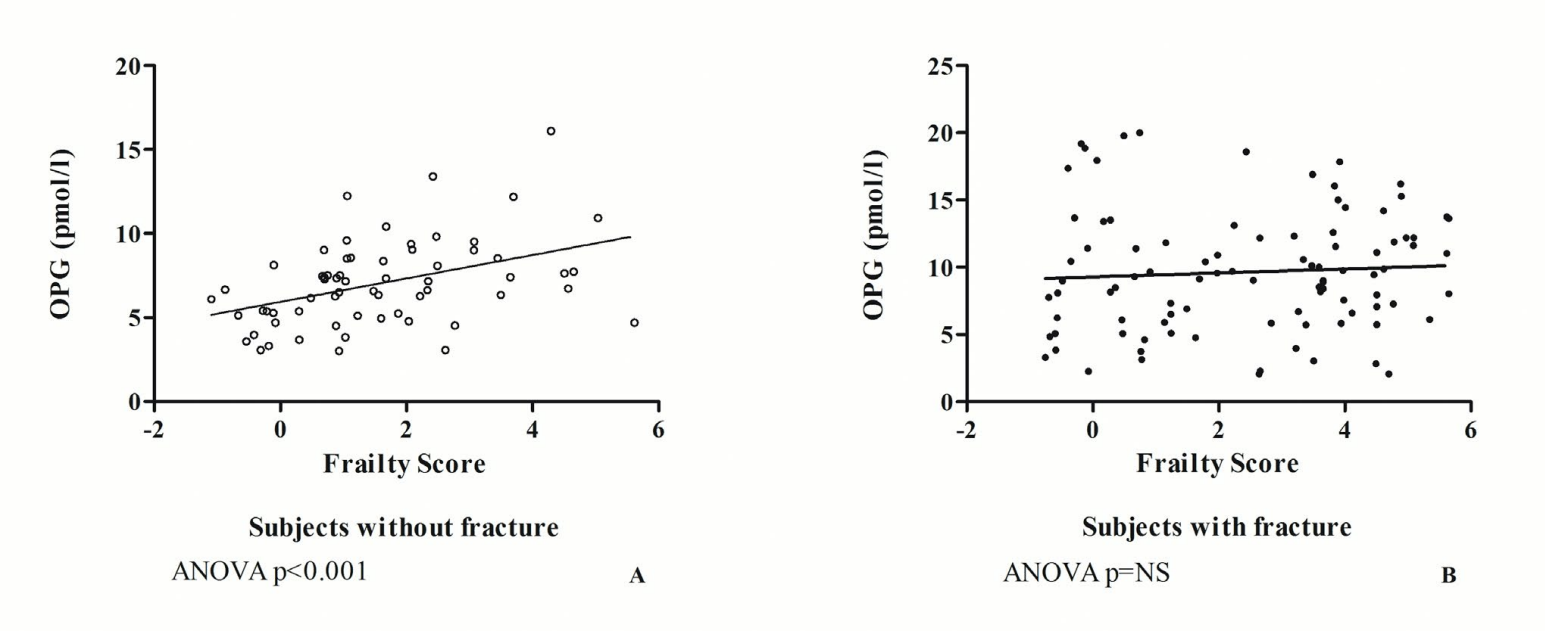
The relationship between OPG and frailty in subjects without fracture (**A**) and in patients with hip fracture (**B**).

As shown in Figure 2, when analyzing separately the data of subjects without fracture from those of patients with hip fracture, it is observed that in the former group (Figure 2 A), there is a statistically significant correlation between OPG and Frailty Score values (ANOVA r =0.420; p<0.001), while in the latter group (Figure 2 B) the two variables are not correlated with each other (r=0.092; p=NS).

**Figure 3:**
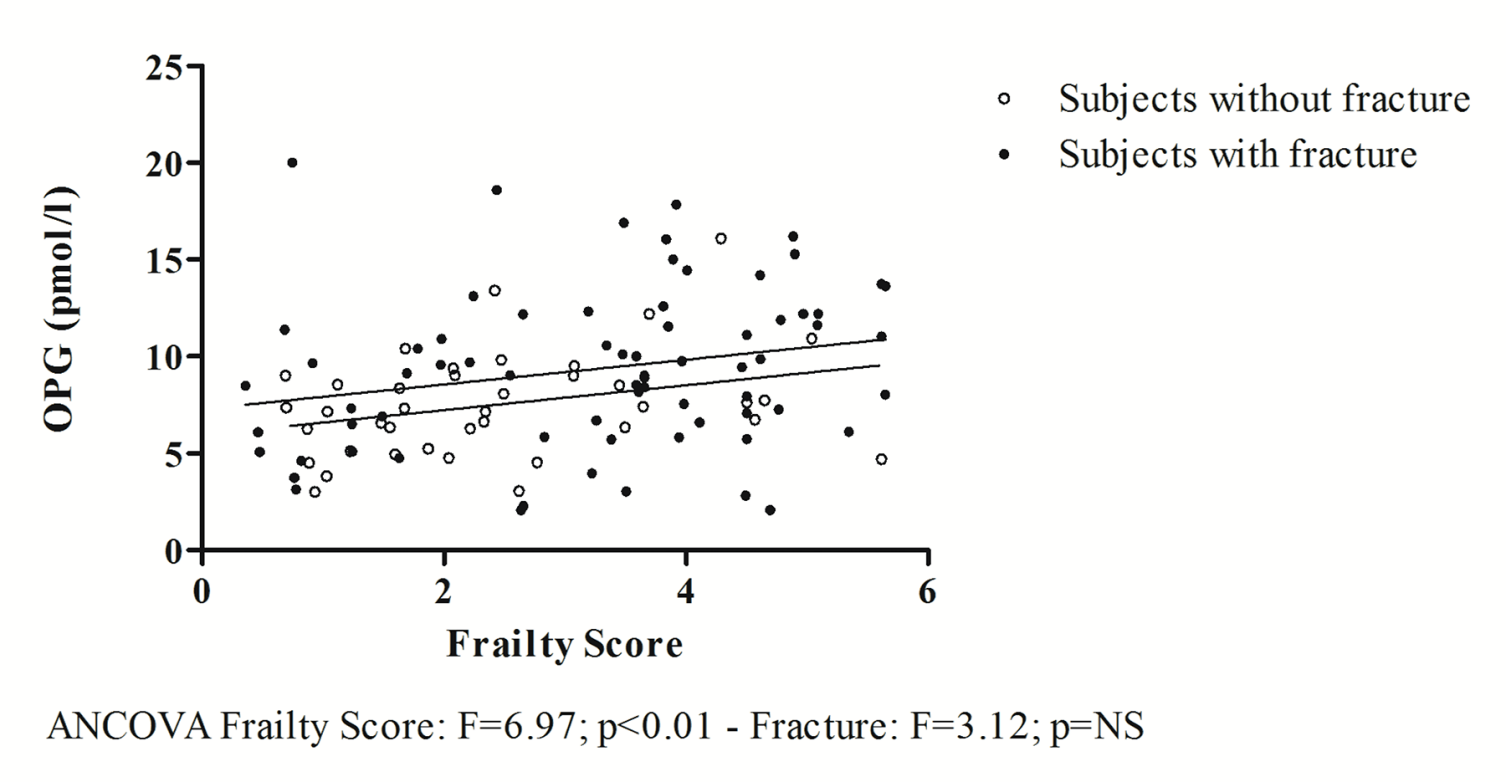
**The relationship between OPG and frailty obtained excluding non-frail subjects.**

A covariance analysis (ANCOVA) excluding non-frail subjects shows that the relationship between OPG and Frailty Score, regardless of fracture (F=3.12; p=0.0804), remains significant in both groups (F=6.97; p<0.01), with a parallel and higher regression line in patients with hip fracture (Figure 3).

**Figure 4:**
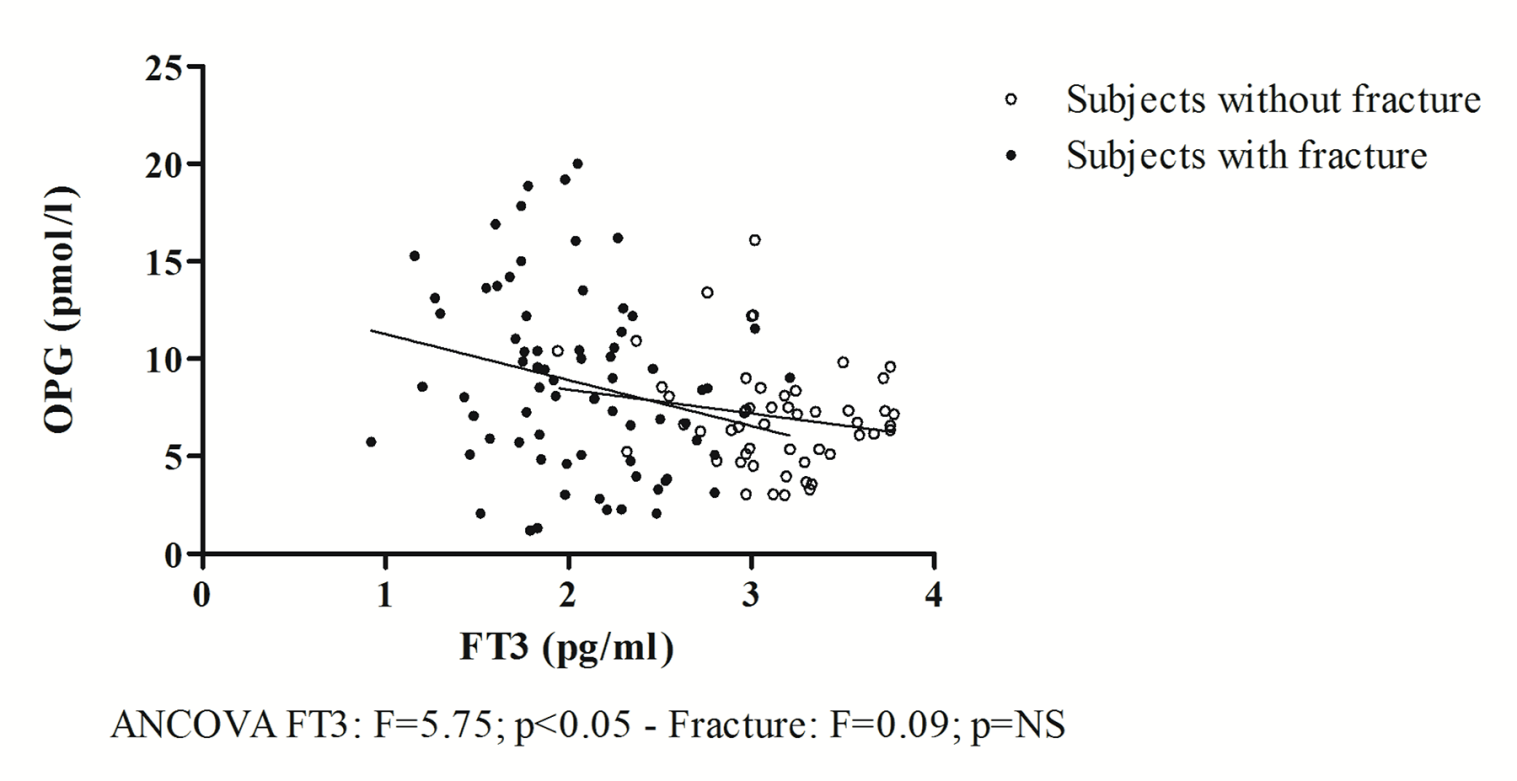
**The relationship between OPG and FT3.**

In the study population, Frailty Score values are inversely associated with FT3 values (ANCOVA F=10.02; p<0.005) regardless of fracture event (ANCOVA F=0.12; p=NS). Furthermore, as shown in Figure 4, FT3 values are inversely and significantly correlated with OPG values (ANCOVA F=5.75, p<0.05), regardless of the presence of fracture (ANCOVA F=0.09; p=NS).

A multivariate linear regression model that includes common laboratory tests (Model 1) was developed, featuring as independent variables FT3, OPG, MCV, WBC, blood glucose, creatinine, albumin, total cholesterol and plasma concentrations of sodium, potassium, calcium and phosphorus. This model showed that, regardless of fracture event, inserted as a covariate, the Frailty Score, inserted as a dependent variable, was associated only with FT3 (p<0.05; CI (95%) -1.758 - -0.015) and with OPG (p<0.05; CI (95%) 0.002 - 0.208). Another multivariate linear regression model, mainly oriented towards endocrine evaluation, (Model 2), which included FT3, OPG, IGF-1, SHBG, cortisol and DHEAS as independent variables, showed that, regardless of fracture event, the Frailty Score was associated with FT3 (p<0.05; CI (95%) -1.820 - -0.191) and OPG (p<0.05; CI (95%) 0.009 - 0.191). Excluding patients with femur fractures, yet another linear multivariate regression model (Model 3) that considered the inflammatory proteins (hs-CRP, IL-6, TNF-α, cortisol) and OPG as independent variables showed that the Frailty Score was predicted by OPG alone (p<0.0001; CI (95%) 0.127 - 0.467).

## DISCUSSION

In the entire study population, the prevalence of frailty was equal to 43.13% and was greater, in both sexes, among patients admitted for fracture (53.61%), who exhibited a greater degree of frailty, compared to outpatients without fractures (26.98%).

Drawing on extant literature [6], we observed that in our study population, frail subjects were older than pre-frail and non-frail subjects and presented modifications of some laboratory parameters characteristic of frailty. In particular, as shown in Table 1, frail subjects had lower mean values of hemoglobin, albumin, FT3, IGF-1 and IGFBP-3 and higher mean values of IL-6, cortisol, hs-CRP, TNF-α and OPG, compared to pre-frail and non-frail subjects.

The modifications of laboratory parameters observed in our study population confirm some of the data in the literature which indicate that, compared to non-frail subjects, frail subjects generally have higher mean values of IL-6 [28–31] and of plasma cortisol and lower mean values of hemoglobin [29], albumin [32], DHEAS [17,33,34], IGF-1 [34] and FT3 [19]. However, fracture events and resulting hospitalizations in our study population played an important role in influencing the serum concentrations of these laboratory parameters. This was supported by an analysis of the covariance that demonstrated how fractures accentuate the differences between frail and non-frail subjects, with an additive, independent effect.

As shown in Table 1, among the study population, frail subjects had significantly higher values of OPG compared to pre-frail and non-frail subjects. Acute secondary events due to fracture had an additive, independent effect on accentuating the differences between groups, so that patients admitted for fracture had higher mean values of OPG than subjects without fracture.

In our population an increase in OPG circulating levels is associated with several conditions frequently observed in frail subjects. It is related to osteoporosis, as suggested by the correlation with neck femoral T-score, to reduced renal function, as suggested by the correlation with GFR and BUN, and to aging itself, and aging is the main cause of frailty (5, 6, 25, 26, 27).

The relationship between OPG and frailty is further highlighted in the regression in Figure 1, which shows how, in the entire study population, higher Frailty Score values correspond to higher OPG values. The analysis of covariance further underscores the close association between the two variables independently of the fracture event, which, in turn, has an additive effect on the relationship between OPG and frailty.

In fact, as per some authors [35], trauma due to a fracture event induces an increase in the expression of OPG. However, as shown in this article, it does not influence the relationship between OPG and frailty. This leads us to hypothesize that the role of OPG is to limit the damage induced by the inflammatory cytokines of the TNF family, as evidenced by the results obtained by analyzing separately the data of the group of patients hospitalized for fracture from those of patients without fracture (Figure 2 A and B). These results suggest that in the study population, the increase in plasma concentrations of OPG has a double origin. In subjects without fracture, OPG, which is well correlated with the Frailty Score, reflects the impairment of various organs and systems that contribute to determining frailty. By contrast, in subjects with hip fracture, OPG produced in response to frailty is supplemented by a share of OPG of bone origin. The production of the latter also depends on the reactive abilities of a subject, which can be greater in non-frail subjects than in pre-frail and frail subjects, as suggested by the loss of correlation between OPG and Frailty Score in subjects with fracture (Figure 2 B).

To further validate the above findings, Figure 3 shows how, by excluding non-frail subjects from the analysis, the correlation between OPG and Frailty Score is statistically significant both in patients with and without fracture. The two regression lines are parallel to each other, but the intercept is greater in patients with hip fracture. This indicates that the fracture event has an additive effect on the increase in plasma concentrations of OPG, which are higher in patients with fractures, but is independent of the relationship between OPG and Frailty Score. Moreover, in our study population, regardless of fracture event, OPG was inversely correlated with FT3 values (Figure 4), whose low concentrations were previously proposed as a possible indicator of frailty in the elderly [19] and of mortality [36].

The close relationship between OPG, FT3 and Frailty Score was confirmed in two multivariate linear regression models where the dependent variable and the covariate were represented by the Frailty Score and the fracture event, respectively; the independent variables were represented in Model 1 by FT3, OPG, MCV, WBC, blood glucose, creatinine, albumin, total cholesterol and plasma concentrations of sodium, potassium, calcium and phosphorus, while in Model 2 they were represented by FT3, OPG, IGF-1, SHBG, DHEAS and cortisol. Both models showed that, independently of fracture events, the Frailty Score was significantly associated only with OPG and FT3 values. This confirms the role of FT3 as a biological indicator of frailty and suggests an analogous role for OPG. In a third model of linear multivariate regression (Model 3), we inserted OPG and the biological indicators of inflammation as independent variables. As these are significantly modified by the acute event, we excluded from the analysis patients with hip fractures. OPG was observed to be the only predictive variable of frailty in this model.

These results led us to two hypotheses. Firstly, the relationship observed between OPG and Frailty Score could reflect the non-specific damaged condition of numerous organs and apparatuses. In non-frail individuals, this could reflect the physiological ageing process, whereas in pre-frail and frail subjects it could be the expression of the progressive accumulation of molecular lesions, cellular and, subsequently, tissutal and organic [37]. These lead to a progressively greater functional impairment manifested in the increase in vulnerability to acute events that characterizes frailty. Secondly, the relationship observed between FT3 and the Frailty Score could be the consequence of the non-specific damage of numerous organs and apparatuses that induces a deterioration of the overall condition of the body, requiring a reduction of energy metabolism [38], thereby configuring low T3 syndrome. Accordingly, the association of frailty and a reduction of FT3/FT4 ratio, a surrogate marker of peripheral thyroxin deiodination, has been recently reported by Pasqualetti G and co-workers [39].

Our data confirm the role of low FT3 values as a biological marker of frailty in the elderly and add new evidence on the role of OPG. Increased levels of OPG are a valid sign of the presence of damage to organs or systems that render elderly people frail. The significant correlation between OPG and Frailty Score found in our study points to its potential use as a biomarker for geriatric frailty syndrome.

## MATERIALS AND METHODS

Our data derive from the extension of an observational study [19] conducted at the "Tor Vergata" University Hospital in Rome on 172 subjects aged 65 or over, whose objective was to identify the main indicators of frailty and to evaluate their relationship with changes of the endocrine system observed with ageing, as described in Bertoli et al [19].

The study population included 107 elderly subjects, representative of a population with a greater prevalence of frailty, having been hospitalised at the Department of Orthopedics and Traumatology for hip fracture which resulted from low-energy trauma, and 65 older subjects with no history of low-energy trauma fracture, who had been referred to the Geriatric Outpatient and Day Hospital Medical Center of the Atherosclerosis Center, as previously described in Bertoli et al [19]. Patients with a history of current or previous malignancies were excluded [19].

The study was approved by the local Ethics Committee and all investigations were carried out in accordance with the principles of the Helsinki Declaration as amended in 2000.

Informed written consent was obtained from each subject who took part in the study and, in the case of patients with mild impairment of cognitive function, informed consent was obtained with the help of a caregiver. All subjects affected by severe impairment of cognitive status and thus not able to understand the objectives of the study and express their consent were automatically excluded.

Anamnestic data and anthropometric parameters (body weight and height) were recorded for each patient, a multidimensional geriatric evaluation including Activity of Daily Living (ADL) and Instrumental Activity of Daily Living (IADL), Mini Mental State Examination (MMSE), Geriatric Depression Scale (GDS), Mini Nutritional Assessment (MNA) and comorbidities assessed using the Cumulative Illness Rating Scale for Geriatrics (CIRS-G) were undertaken as described in previous work [8], and blood samples measured blood count, blood glucose, glycosylated hemoglobin (HbA1c), creatinine, Thyroid Stimulating Hormone (TSH), Free Triiodothyronine (FT3), Free Thyroxine (FT4), Parathyroid Hormone (PTH), 25-hydroxyvitamin D3 (25OHD), osteoprotegerin, cortisolemia, Dehydroepiandrosterone Sulfate (DHEAS), Sex Hormone Binding Globulin (SHBG), Growth Hormone (GH), Insulin-like Growth Factor-1 (IGF-1), Insulin-like Growth Factor Binding Protein-3 (IGFBP-3), Inteleuchin-6 (IL-6), Tumor Necrosis Factor-α (TNF-α), and high sensitivity-C Reactive Protein (hs-CRP).

The degree of frailty was calculated using the Survey of Health, Ageing and Retirement in Europe Frailty Instrument (SHARE-FI) [16].

The blood count was evaluated by routine laboratory tests (Sysmex XE 2100, Dasit). Blood glucose and creatinine levels were measured using homogeneous chemiluminescence assay (Dimension VISTA 1500, Siemens). HbA1c was assessed using capillary electrophoresis (Capillarys 2, Sebia), OPG was measured using an immunoenzymatic method (Biomedia), plasma concentrations of PTH, 25OHD, TSH, FT3, FT4 and cortisolemia were measured using chemiluminescence assay (ADVIA Centaur XP, Siemens). ACTH, DHEAS, SHBG, GH, IGF-1 and IGFBP-3 were measured with chemiluminescence assay (Immulite 2000, Siemens). hs-CRP was assessed with a nephelometric method (Dimension Vista 1500, Siemens), while IL-6 and TNF-α were measured using the immuno-reflex method (DRG). The degree of frailty of each patient was calculated using the Frailty Instrument for Primary Care of the Survey of Health, Ageing and Retirement in Europe (SHARE-FI) [16], based on the main identification criteria of frailty introduced by Fried LP et al [6]. Muscle strength was measured as muscle-holding force of the dominant hand using a Jamar-like digital hand-held dynamometer (Kern & Sohn, Balingen, Germany), according to which normal values for the elderly population are identified as greater than 30 Kg for male subjects and above 20 Kg for female subjects [40].

### Statistical analysis

The Kolmogorov-Smirnov test was used to verify the normal distribution of parameters.

For comparison between the groups, the Student’s *t*-test and the analysis of variance (ANOVA) were used. Correlations were evaluated by simple linear regression. Multivariate linear regression was used to estimate the predictive variables of frailty. Statistical analysis was performed using Stat View 5 (SAS Institute, Cary North Carolina, USA) and SPSS 21 (IBM SPSS Statistics, Version 21.0. Armonk, NY: IBM Corp. IBM). The graphs were developed using GraphPad Prism 5 (GraphPad Software Inc., San Diego, California, USA).

Data are presented as mean ± standard deviation.

Values of p<0.05 were considered significant.
